# Revisiting the *Raractocetus* Fossils from Mesozoic and Cenozoic Amber Deposits (Coleoptera: Lymexylidae)

**DOI:** 10.3390/insects13090768

**Published:** 2022-08-25

**Authors:** Yan-Da Li, David Peris, Shûhei Yamamoto, Yun Hsiao, Alfred F. Newton, Chen-Yang Cai

**Affiliations:** 1 State Key Laboratory of Palaeobiology and Stratigraphy, Nanjing Institute of Geology and Palaeontology, Centre for Excellence in Life and Palaeoenvironment, Chinese Academy of Sciences, Nanjing 210008, China; 2 School of Earth Sciences, University of Bristol, Life Sciences Building, Tyndall Avenue, Bristol BS8 1TQ, UK; 3 Departament de Dinàmica de la Terra i de l’Oceà, Facultat de Ciències de la Terra, Universitat de Barcelona, 08028 Barcelona, Spain; 4 Institut de Recerca de la Biodiversitat (IRBio), Universitat de Barcelona, 08028 Barcelona, Spain; 5 Hokkaido University Museum, Hokkaido University, Kita 10, Nishi 8, Kita-ku, Sapporo 060-0810, Japan; 6 Australian National Insect Collection, Commonwealth Scientific and Industrial Research Organisation, GPO Box 1700, Canberra, ACT 2601, Australia; 7 Division of Ecology and Evolution, Research School of Biology, Australian National University, Canberra ACT 2601, Australia; 8 Negaunee Integrative Research Center, Field Museum of Natural History, 1400 S Lake Shore Drive, Chicago, IL 60605, USA

**Keywords:** Atractocerinae, *Cretoquadratus*, Kachin amber, ambrosia beetle

## Abstract

**Simple Summary:**

Lymexylidae is a small beetle family, with some members exhibiting strongly reduced elytra and largely exposed functional hind wings. Previously, four species from Kachin (Myanmar), Baltic, and Rovno ambers were assigned to the extant lymexylid genus *Raractocetus*. Our new examination suggests that these fossils are morphologically separated from the extant *Raractocetus*, primarily in the hind wing venation, and should be removed from *Raractocetus*.

**Abstract:**

The fossils once assigned to *Raractocetus* Kurosawa from the Mesozoic and Cenozoic amber deposits differ from extant *Raractocetus* in the longer elytra, the more strongly projecting metacoxae, and the hind wing with vein 2A forked. Thus, these fossils should be removed from *Raractocetus*. *Cretoquadratus engeli* Chen from Kachin amber appears to be conspecific with *R.* *fossilis* Yamamoto. As a result, *R. fossilis* and *R. extinctus* Yamamoto from Kachin amber, *R. balticus* Yamamoto from Baltic amber, and *R. sverlilo* Nazarenko, Perkovsky & Yamamoto from Rovno amber are transferred to *Cretoquadratus* Chen, as *C.* *fossilis* (Yamamoto) **comb. nov.**, *C. extinctus* (Yamamoto) **comb. nov.**, *C. balticus* (Yamamoto) **comb. nov.,** and *C. sverlilo* (Nazarenko, Perkovsky & Yamamoto) **comb. nov.**, and *C. engeli* **syn. nov.** is suggested to be a junior synonym of *C. fossilis*.

## 1. Introduction

With 84 worldwide distributed species in 19 genera (including fossils), Lymexylidae is divided into four subfamilies: Atractocerinae (nine genera, with three extinct), Hylecoetinae (one genus), Lymexylinae (one genus), and Melittommatinae (five genera, with one extinct) [1,2,3,4,5,6,7]. In addition, one fossil genus was unable to be confidently assigned to any existing subfamily [8,9].

Historically, Lymexylidae had once been associated with Cleroidea or Cucujoidea [10,11], but molecular studies have recovered an affinity with Tenebrionoidea. In three of the studies, Lymexylidae appeared to be nested within basal Tenebrionoidea, with various positions [12,13,14], although only a few gene fragments were sampled in these studies. Other studies, including three recent phylogenomic ones, suggested Lymexylidae as the sister group of (the remaining) Tenebrionoidea [15,16,17,18,19]. Wheeler [20] concluded that the maxillary palporgan is the strongest autapomorphy for this family, and considered its loss in *Australymexylon* Wheeler as secondary.

The fossil record of Lymexylidae is relatively abundant. Many lymexylid fossils have been recently reported from several Mesozoic and Cenozoic deposits. Almost all known fossils from the family are from amber deposits [3,4,5,6,7,8,21]. As resiniferous deposits seem to entrap arthropod fauna living primarily in or near the resiniferous trees [22], a potential relationship between the family and gymnosperm trees, the source of Kachin, Baltic and Rovno ambers [23,24,25,26], can be postulated, given the close developmental association of modern lymexylids with trees (see Section 4).

Four species of fossil lymexylids from Mesozoic and Cenozoic deposits were assigned to the extant genus *Raractocetus* Kurosawa [5,6]. However, these *Raractocetus* fossils differ from extant members of *Raractocetus* in having longer elytra and more strongly projecting metacoxae. In this study, we examine additional specimens of *R. fossilis* Yamamoto from mid-Cretaceous Kachin amber, which provide new clues for the systematic placement of these *Raractocetus* fossils.

## 2. Materials and Methods

**Figure 1:**
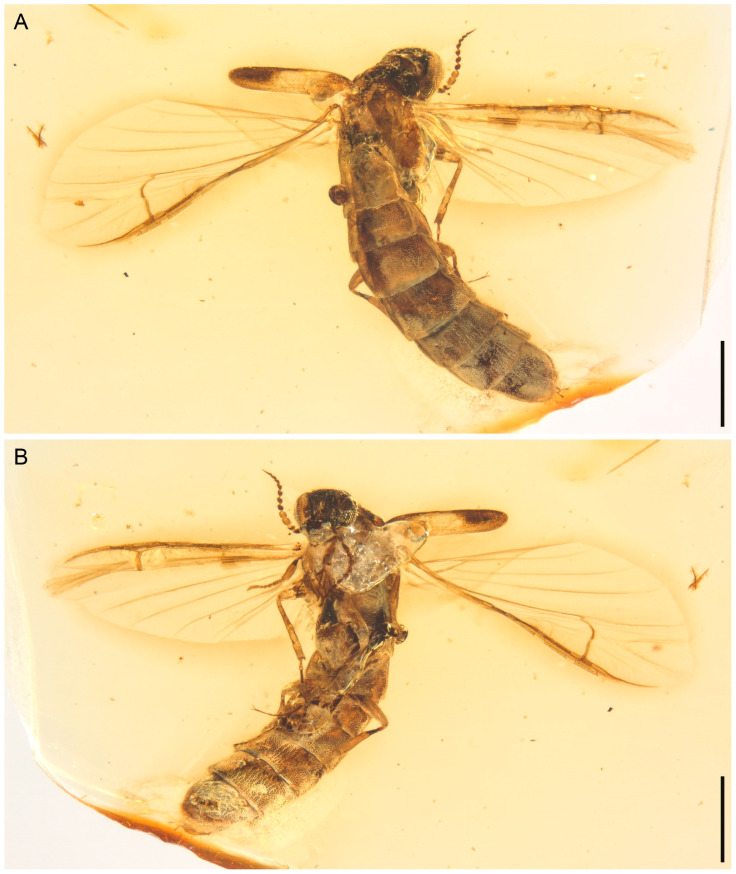
General habitus of *Cretoquadratus fossilis* (Yamamoto) **comb. nov.**, NIGP180657, under incident light. (**A**) Dorsal view. (**B**) Ventral view. Scale bars: 1 mm.

**Figure 2:**
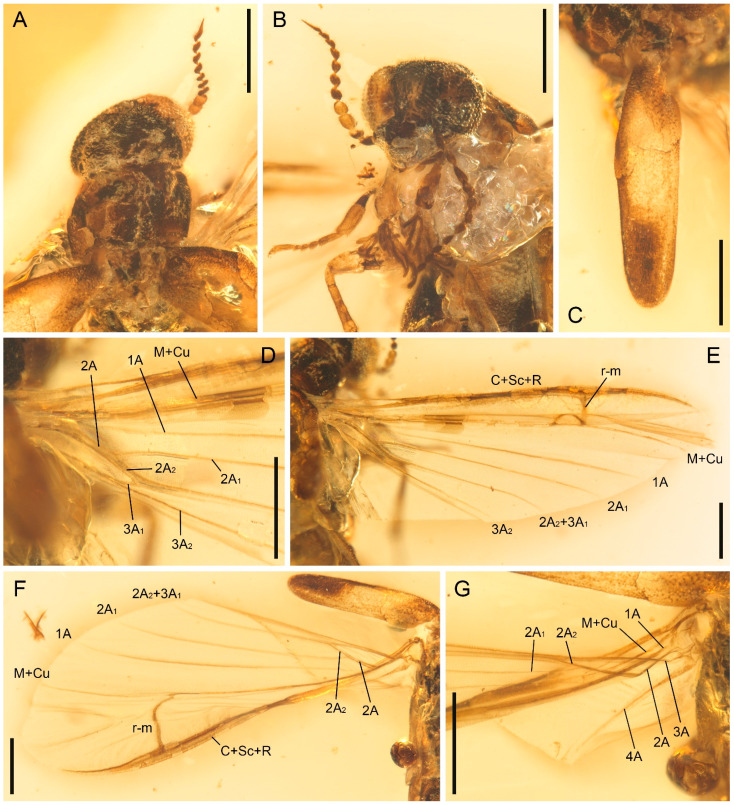
Details of *Cretoquadratus fossilis* (Yamamoto) **comb. nov.**, NIGP180657, under incident light. (**A**) Head and prothorax, dorsal view. (**B**) Head, anterior view. (**C**) Elytron, dorsal view. (**D**,**E**) Right hind wing. (**F**,**G**) Left hind wing. Scale bars: 500 μm.

**Figure 3:**
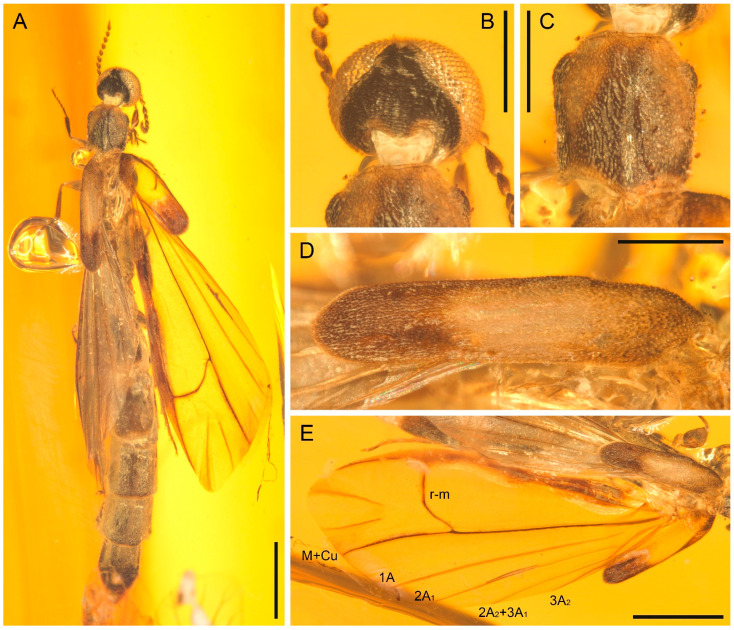
*Cretoquadratus fossilis* (Yamamoto) **comb. nov.**, NIGP180658, under incident light. (**A**) Habitus, dorsal view. (**B**) Head, dorsal view. (**C**) Prothorax, dorsal view. (**D**) Elytron, dorsal view. (**E**) Right hind wing. Scale bars: 1 mm in (**A**,**E**), 400 μm in (**B**–**D**).

**Figure 4:**
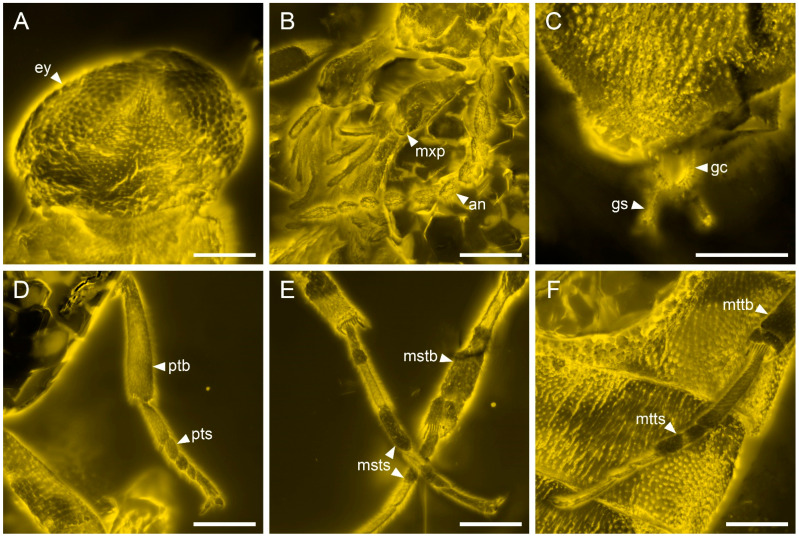
Details of *Cretoquadratus fossilis* (Yamamoto) **comb. nov.**, NIGP180657, under confocal microscopy. (**A**) Head, dorsal view. (**B**) Antenna and maxillary palps. (**C**) Ovipositor, dorsal view. (**D**) Fore leg. (**E**) Mid legs. (**F**) Hind leg. Abbreviations: an, antenna; ey, compound eye; gc, gonocoxite; gs, gonostylus; mxp, maxillary palp; mstb, mesotibia; msts, mesotarsus; mttb, metatibia; mtts, metatarsus; ptb, protibia; pts, protarsus. Scale bars: 200 μm.

The new specimens of *Cretoquadratus fossilis* (Yamamoto) (NIGP180657 and NIGP180658; Figure 1, Figure 2, Figure 3 and Figure 4) originated from amber mines near Noije Bum (26°20′ N, 96°36′ E), Hukawng Valley, Kachin State, northern Myanmar, and are deposited in the Nanjing Institute of Geology and Palaeontology (NIGP), Chinese Academy of Sciences, Nanjing, China. Amber pieces containing the new specimens were trimmed with a small table saw, ground with emery paper of different grit sizes, and finally polished with polishing powder.

For a comparative purpose, S.Y. examined the holotypes of *Raractocetus fossilis* Yamamoto, *R. extinctus* Yamamoto, and *R. balticus* Yamamoto, which are deposited in the Gantz Family Collections Center, Field Museum of Natural History (FMNH), Chicago, IL, USA; Y.H. examined specimens of extant *R. kreusleri* (Pascoe), which are deposited in the Australian National Insect Collection (ANIC), Commonwealth Scientific and Industrial Research Organisation (CSIRO), Canberra, Australia.

Photographs under incident light were taken with a Zeiss Discovery V20 stereo microscope. Confocal images were obtained with a Zeiss LSM710 confocal laser scanning microscope, using the 561 nm (DPSS 561-10) laser excitation line [27]. Images under incident light were stacked in Helicon Focus 7.0.2. Confocal images were semi-manually stacked with Helicon Focus 7.0.2 and Adobe Photoshop CC. Images were further processed in Adobe Photoshop CC to adjust brightness and contrast.

The terms for hindwing venation follow Wheeler [20], without regard to whether they are anatomically correct.

This published work has been registered in ZooBank, the official registry of Zoological Nomenclature. The LSID for this publication is urn:lsid:zoobank.org:pub:16BAE184-E1DE-4976-A0DB-68698CED438C.

## 3. Systematic Palaeontology

Order Coleoptera Linnaeus, 1758
Suborder Polyphaga Emery, 1886
Superfamily Lymexyloidea Fleming, 1821
Family Lymexylidae Fleming, 1821
Subfamily Atractocerinae Laporte, 1840
**Genus *Cretoquadratus* Chen, 2019**
**Type species.** *Cretoquadratus engeli* Chen, 2019.

**Composition.** *Cretoquadratus fossilis* (Yamamoto, 2019) **comb. nov.** (=*Cretoquadratus engeli* Chen, 2019 **syn. nov.**), *Cretoquadratus extinctus* (Yamamoto, 2019) **comb. nov.**, *Cretoquadratus balticus* (Yamamoto, 2019) **comb. nov.**, *Cretoquadratus sverlilo* (Nazarenko, Perkovsky & Yamamoto, 2022) **comb. nov.**

**Revised diagnosis.** Eyes large, occupying almost entire frons, nearly contiguous anteriorly (Figure 2A,B, Figure 3B, and Figure 4A; Yamamoto [5]: supplementary Figures S4b,c, S6c,e and S8c,d; Yamamoto et al., 2022: Figure 4A,C). Antennae more or less fusiform (Figure 2 and Figure 4B; Yamamoto [5]: supplementary Figures S7b and S8e; Yamamoto et al. [6]: Figure 3A). Pronotal disc subquadrate or slightly elongate (Figure 3A; Yamamoto [5]: supplementary Figures S4 and S8b; Chen [4]: Figure 1.3). Elytra reduced, but relatively oblong, each more than three times as long as wide (Figure 2C and Figure 3D; Yamamoto [5]: Figure 2g–i; Yamamoto et al. [6]: Figure 4D). Hind wings with M+Cu fork absent, 1A unforked, and 2A forked (Figure 2D–G and Figure 3E; see also Yamamoto et al. [6]: Figure 2). Metacoxae relatively strongly projecting posteriorly (Yamamoto [5]: supplementary Figures S5b, S7a and S9b; Yamamoto et al. [6]: Figure 3D). Tibial spurs 0-0-0 in male [4], 0-1-1 in female (Figure 4D–F; see also [5]).

**Remarks.** Yamamoto [5] and Yamamoto et al. [6] reported several fossils of Atractocerinae from Mesozoic and Cenozoic amber deposits. Among them, four species were assigned to the extant genus *Raractocetus*, i.e., *R. fossilis* and *R. extinctus* from Kachin amber, *R. balticus* from Baltic amber, and *R. sverlilo* from Rovno amber of Ukraine. *Raractocetus* is closely related to *Atractocerus* Palisot de Beauvois, and contains only two extant species [28]. As characterized by Paulus [1], extant *Raractocetus* (and also *Atractocerus*) have very short elytra, which are only slightly longer than wide, and non-projecting metacoxae, which are not longer than the trochanters. However, the fossil species assigned to *Raractocetus* have longer elytra and more strongly projecting metacoxae [5,6]. Our new specimens of *R. fossilis*, NIGP180657 and NIGP180658, further provide additional information on the hind wing venation. Extant *Raractocetus* has hind wing venation essentially equal to *Atractocerus*, where vein 1A is forked (e.g., Wheeler [20]: Figures 127, 128; Casari & Albertoni [29]: Figure 48), whereas in *R. fossilis* vein 1A is unforked and, instead, vein 2A is forked (Figure 2D–G and Figure 3E). As such, it is well justified to place those fossil species formerly placed in *Raractocetus* in a separate genus.

Chen [4] reported another fossil from Burmese amber, which he assigned to a new genus and species, i.e., *Cretoquadratus engeli*. The most important character of *Cretoquadratus* claimed by Chen [4] is the presence of media branches M1–M4 originated from the cross point of the r-m and M+Cu veins, which, however, are not visible in the left hind wing (Chen [4]: Figure 1). Thus, we suggest that such media branches M1–M4 seemingly present on the right wing of *C. engeli* are likely to be artifacts, and should not be regarded as a diagnostic character. The paired longitudinal ridges on the scutellar shield (mesonotum actually; see [30]), suggested by Chen [4] as an additional diagnostic character for *Cretoquadratus*, are commonly present in the subfamily Atractocerinae, including the fossil and extant *Raractocetus*. Other character of *Cretoquadratus*, for example, the approximation of eyes and the shape of head and pronotum, are well comparable with the fossils assigned to *Raractocetus*. In fact, the body size, the antennal structure, the pronotal shape, and the darkened elytral apex of *C. engeli* are essentially identical to those of *R. fossilis*; therefore, we believe that *C. engeli* is conspecific with *R. fossilis*. Both of these names were (likely) first published in 2019, but according to their publishers, Yamamoto [5] was published on 20 March 2019, while Chen [4] was published no earlier than December 2019. Thus, *C. fossilis* has priority over *C. engeli* as the valid name of this species.

## 4. Discussion

In this study, we report the additional materials of “*Raractocetus*” *fossilis* from mid-Cretaceous Kachin amber from northern Myanmar. Our materials reveal several additional important character, such as wing veins, which allow further discussion on the generic identity with the recently described similar atractocerine taxa from both Mesozoic and Cenozoic deposits. Based on our new morphological data, with re-observation of some of the previously described material, we propose a total of four new generic combinations and a junior synonymy. As a result, the mid-Cretaceous genus *Cretoquadratus* is now known from not only the Mesozoic but also from the Cenozoic (Eocene), which indicates that *Cretoquadratus* successfully survived the K-Pg mass extinction, but likely went extinct later and does not last until the present day. This finding also reinforces the idea that the genus was once widely distributed, based on its presence in various fossil localities.

Larvae of all known lymexylid species develop in decaying wood or tree trunks [31]. This wood-boring habit of larvae and their feeding primarily on symbiotic fungi cultivated inside the galleries [20,31,32,33] is what led Peris et al. [34] to suggest that Lymexylidae might actually be considered as ambrosia beetles, following the criteria by Kirkendall et al. [35]. The estimated origin for the earliest groups of ambrosia fungi during the Cretaceous period concurs with a known high diversity of Lymexylidae from the amber record [34,36], which has been suggested to have an Early Jurassic or even Triassic origin based on molecular dating [18,19]. By contrast, more typical ambrosia beetles, Scolytinae and Platypodinae (Curculionidae), radiated later (although the earliest scolytine fossil is known from the Early Cretaceous [37]), developing a fungus-farming behavior associated with angiosperms during the Late Cretaceous [38], or perhaps later [34]. This fact points to lymexylids as potential early vectors of ambrosia fungi since the Early Cretaceous, before the close fungal relationship with Scolytinae and Platypodinae evolved [34]. However, the direct impact of wood-borer or ambrosia beetles in resin production during the Cretaceous has not yet been properly demonstrated [36,39] and is a question that needs further attention.

## Data Availability

The original confocal data are available on the Zenodo repository (doi:10.5281/zenodo.7008334).
